# Eco-epidemiology of visceral leishmaniasis in Ethiopia

**DOI:** 10.1186/s13071-015-0987-y

**Published:** 2015-07-19

**Authors:** Endalamaw Gadisa, Teshome Tsegaw, Adugna Abera, Dia-eldin Elnaiem, Margriet den Boer, Abraham Aseffa, Alvar Jorge

**Affiliations:** Armauer Hansen Research Institute, Addis Ababa, Ethiopia; Department of Natural Sciences, University of Maryland Eastern Shore (for KalaCORE consortium), 1Backbone Rd, Princess Anne, MD 21853 USA; Visceral Leishmaniasis Program, Drugs for Neglected Diseases Initiative (DNDi), Addis Ababa, Ethiopia; KalaCORE consortium, Backbone Rd, Princess Anne, MD 21853 USA

## Abstract

Visceral leishmaniasis (VL, Kala-azar) is one of the growing public health challenges in Ethiopia with over 3.2 million people at risk and estimated up to 4000 new cases per year. Historically, VL was known as the diseases of the lowlanders; in the lower and upper Kola agro-ecological zones of Ethiopia. The 2005–07 out breaks in highlands of Libo Kemkem and Fogera, in the Woina Degas, that affected thousands and claimed the life of hundreds misdiagnosed as drug resistance malaria marked that VL is no more the problem of the lowlanders. The Kola (lower and upper) and the Woina Dega are the most productive agroecological zones, supporting both the ongoing and planned expansions of large or small scale agriculture and/or agriculture based industries. Thus, the (re)emergence of VL is not only a public health and social problem but also have a direct implication on the country’s economy and further development. Thus is high time for its control and/or elimination. Yet, the available data seem incomplete to plan for a cost-effective and efficient VL control strategy: there is a need to update data on vector behaviour in specific ecosystems and the roles of domestic animals need to be ascertained. The effectiveness and social acceptability of available vector control tools need be evaluated. There is a need for identifying animal reservoir(s), or establish the absence of zoonosis in Ethiopia. The planning of prevention of (re)emergence and spread of VL to areas adjacent to endemic foci need be supported with information from spatio-temporal mapping. In affected communities, available data showed that their knowledge about VL is generally very low. Thus, well designed studies to identify risk factors, as well as better tools for social mobilization with the understanding of their knowledge, aptitude and practice towards VL are necessary.

## Introduction

Visceral leishmaniasis (VL or kala-azar) is a (re)emerging [1] neglected tropical disease, endemic in over 79 countries [2, 3]. Diagnosis and treatment of VL is difficult and without appropriate treatment an estimated 95 % of VL patients will die. The disease may kill between 20,000 and 40,000 people per year globally. Of those infected, a large majority remain asymptomatic. The subclinical to clinical case ratio ranges from 1:1.2 to 1:9 depending on the eco-epidemiological situation [4–6]. Over 90 % of the annual incidence, 0.2 to 0.4 million new cases per year, occur in Bangladesh, India, Nepal, Sudan, South Sudan, Ethiopia and Brazil. Eastern Africa, the second largest VL focus after the Indian subcontinent, contributes to the global burden with 30,000–40,000 new cases per year, of which Ethiopia, South Sudan and Sudan contributing the largest proportion of the cases [2]. In Ethiopia an estimated 2500 to 4000 new cases occur per year and over 3.2 million people are at risk of infection [7, 8].

The recorded history of VL in Ethiopia dates back to the 1940s with reports of VL cases among members of the East African British Army deployed along the Ethiopian-Kenyan border during the Second World War [9]. In their report on the outbreak of kala-azar in the battalion of the King’s African Rifles, Cole et al. [10] documented that among confirmed cases of leishmaniasis, half (10/22) probably contracted the disease in the low lying Omo River area, in the south-western corner of Ethiopia. Subsequent field and longitudinal studies confirmed the endemicity of VL in Ethiopia [11–14]. The vernacular name in Konso language, ‘golloba’, referred to in the retrospective study by Lindtjorn (1970–1981) indicates that the disease was already well known in the communities of the Segen and Woyto valley before World War Two [12].

**Fig. 1:**
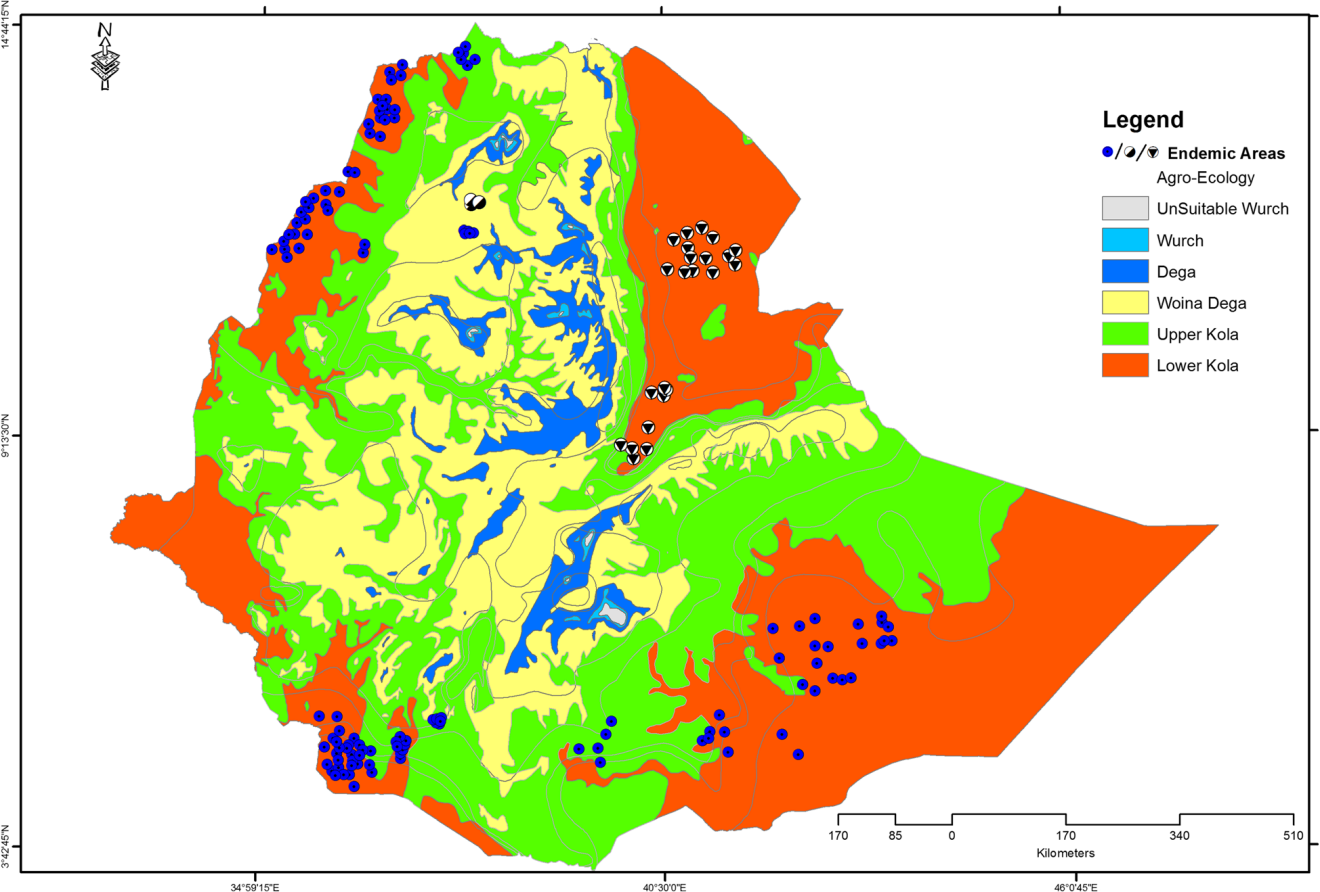
Endemic foci of visceral leishmaniasis (VL) in Ethiopia. VL occurs mainly in the Kola agro-ecological zones with recent spread to the Woina degas of Libo Kemkem and Fogera. The circles summarize endemic foci so far described: confirmed human cases from autochthonous transmission (blue circles); doubtful: no evidence of VL cases from autochthonous transmission (circles with central black triangle); or seroprevalence only with no human clinical VL (half black circles)

The VL foci from which clinical cases are reported differ in their eco-epidemiology and the sandfly vectors involved. Currently, VL transmission is known to occur in three different ecological settings, spanning from lower Kola to the Woina Dega ecological zones (Fig. 1). However, VL seems not to have been established despite the presence of the appropriate vectors and high reported leishmanin skin test positivity and/or sero-prevalence along the course of the Awash valley [15–17] and in the Gambella region [18].

Importantly affected by altitude, Ethiopia’s climate is mainly of the tropical monsoon type with wide topography-induced variation. Classified by topography and thermal and moisture regimes (from over 20 year (1974 to 1993) ground data on major rain-fed agricultural crops), Ethiopia has six agro-ecological zones: Unsuitable Wurch, Wurch, Dega, Woina Dega, Upper Kola and Lower Kola [19]. In terms of geographical extent, 0.14 % (1187.34 km^2^), 0.33 % (2870.83km^2^), and 5.09 % (43,943.81km^2^) of the total landmass belongs to the Unsuitable Wurch, the Wurch and the Dega agro-ecological zones, whilst the major portion of the country, 31.04 % (267,847.95 km^2^), 46.02 % (397,115.47 km^2^), and 17.38 % (149,945.56 km^2^) is covered by the Woina Dega, Upper Kola and Lower Kola zones respectively. Overlaying 20 consecutive years of Ethiopian National Meteorology Agency data (1989 to 2009), the average mean surface temperature for Lower Kola, Upper Kola and Woina Dega is 26.35 °C, 22.05 °C and 17.81 °C, and the average annual rainfall is 490.48 mm, 871.84 mm and 1162.80 mm respectively. When the FAO (2000) soil map is clipped to the country’s boundary and overlaid on the agro-ecologic zones, the five major soil types in a decreasing order are Leptosols, Nitosols, Vertisols, Cambisols and Calsisols in all agro-ecological zones. The major agricultural activities and production of the country occur in the Woina Dega ecological zone whereas large scale private, government and resettlements for both irrigated and rain-fed agriculture are currently being implemented or planned in the upper and lower Kola agro-ecological zones.

### The southern lowland foci

The southern foci are the south-western savannah and the south-eastern semi-arid lowlands which account for approximately 20 % of the total VL burden in Ethiopia [11]. The Omo and Aba-Roba plains and the Woyto and Segen River valleys in the Southern Nations, Nationalities and Peoples’ Regional State (SNNPR) are the oldest known VL foci in Ethiopia [13, 14]. These foci are characterized by lower to upper Kola agro-ecology with an altitude that ranges from 500–1500 m above sea level (masl). The area is inhabited by nomadic or semi-nomadic pastoralist communities. Several studies have described the foci. Leishmanin skin test surveys in the 1970s showed different levels of VL endemicity among the communities in the lower Omo (400–600 masl), upper Omo (1400 masl) and Hamar (~1000 masl) areas with >65 %, 6.4 % and 21.2 % positivity rates respectively [13, 14]. A survey conducted in the 1880s identified the Genale river basin and the western part of Moyale town as endemic areas [11]. A prospective study conducted from 1997 to 2000 in Aba-Roba further established it as the focus with the highest VL endemicity of the south-west [20], and confirmed earlier reports of the spatio-temporal and age differences in the prevalence and incidence of VL in the region [4]. Further eastward, in the Oromia regional state, sporadic VL cases have been reported from Lake Abaya, Moyale area and the Dawa and Genale River valleys [11, 21]. Down in the south-eastern semi-arid lowlands, there is a recent report of VL transmission in the Afder and Liben areas in the Somali regional state [22].

### Northern lowland foci

The Metema and Humera plains in the Tigray and Amhara regional states, bordering Sudan and Eritrea, constitute the main VL endemic area in the country, contributing over 60 % to the burden. These foci are also in the lower and upper Kola agro-ecological zones, with wide, open plains covered in bush scrubs and Acacia woodland. The woodland cover is in process of being replaced by extensive commercial agriculture that produces sesame as the main cash crop. Leishmanin skin test surveys on 1057 participants in Humera in the 1970s among predominantly new settlers (4.4 years average stay) documented a marked difference of prevalence in the farming (45.6 %) and non-farming (8.3 %) communities and showed that overall skin test positivity increased with the duration of stay in the area [23]. A sharp rise in the number of VL cases was attributed to the influx of seasonal temporary workers for the large-scale agricultural schemes and forced resettlements of populations from the neighboring highlands [23–25]. A high HIV prevalence among seasonal workers has contributed to the rise in VL prevalence in this group [26–28]. The highest HIV/VL co-infection rate world-wide (~38 %) was reported in this region. A Médecins Sans Frontières (MSF) treatment centre treated 2000 to 5000 VL cases per year in this area at one point in time [28]. Recently, VL has spread beyond the Metema and Humera plains to Tahtay Adiabo, Welkaite, and Mierab Armacheho in the Tigray and Amhara regional states [7, 29].

### The highland foci

The claim that VL also exists in Ethiopia’s highlands dates back to the 1970s with the narrative of two cases reported in Belessa without a travel history [30]. Yet, the confirmation of autochthonous transmission remains doubtful. Belessa is a highland area (1800 to 2500 masl) with broad plateau expanses of black soil, steep mountain sides, flat topped promontories and deep cuts of river valleys and galleys. Its ecology is distinctly different than the lowland foci. In 2005–07 the adjacent highland (>1800 masl) districts Libo Kemkem and Fogera experienced a large-scale outbreak of VL which affected thousands of people [31, 32]. Initially, VL was misdiagnosed as drug resistant malaria since the disease was not supposed to occur at this altitude. The apparently continued low transmission after the outbreak in Libo Kemkem [33] indicates the potential dispersal and establishment of VL transmission in highlands adjacent to the lowland foci; a growing concern for the upper Kola to Woina Dega ecological zones.

### Vectors

**Fig. 2:**
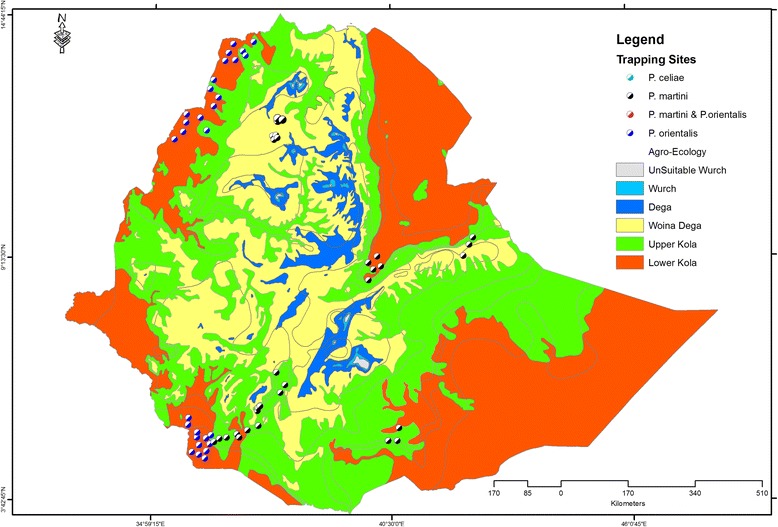
Geographic spread of sandfly vectors in Ethiopia. Current data show that the geographic distribution of the sandfly vector for *L. donovani* seems to extend from the lower Kola to the Weina Dega ecological zones. Circles summarize approximate (data compiled from description of trapping/study site or place/village names or village/community level) coordinates of trapping sites or exact coordinates of the trapping point

Sandflies are limited with respect to the *Leishmania* species they are able to transmit [34]. Of the 42 sandfly species implicated in the Old World, only six transmit *L. donovani* [35]*.* Of these, only *Phlebotomus orientalis* (*P. orientalis*), *P. celiae* [36] and *P. martini* [36] are incriminated as vectors for *L. donovani* in Ethiopia. The transmission is generally assumed to be anthroponotic, yet no evidence exists to refute the early assumption of zoonoses with occasional spill-over to humans [11, 37]. Recent detection of DNA typed as *L. donovani* from wild rodents [38], epidemiological [39, 40] and parasite genetics data [41] actually seem to strengthen the zoonotic claim. However, the reservoir host(s) and vector(s) supporting zoonotic *L. donovani* transmission in Ethiopia remain to be determined. The lack of information on a zoonotic cycle could possibly be due to inadequate range of trapping methods [42] and screening surveys in animal hosts. Documented studies so far showed that the geographic extent of *P. orientalis* and *P. martini* is wider than the known endemic foci of VL (Fig. 2). Thus delineating areas with vector presence from zones endemic for VL is a priority to plan for containment of transmission. The flight range of most sandfly species is typically short (~300 m), while the breeding sites are assumed to be in the vicinity of areas where males with unrotated external genitalia and females with blood meal and egg stages exist [34, 35]. Thus systematic representation of ecological zones with appropriate trapping techniques complemented by GIS based modelling could give adequate information on their habitat preference and/or geographic extent. Overlaying the sandfly vector geographic extent information on the autochthonous VL transmission zone would enable the delineation of areas that are potentially at high risk of introduction of VL.

### *Phlebotomus (Larroussius) orientalis* (Parrot)

*P. orientalis,* one of the principal vectors of *L. donovani* in East Africa, was first described in the Dire-Dawa area of the Oromia regional state of Ethiopia (an area from where no clinical cases of VL have so far been reported). *P. orientalis* has also been reported in Chad, Egypt, Kenya, Niger, Rwanda, Saudi Arabia, South Yemen, Sudan, South Sudan and Uganda [43]. In Ethiopia, *P. orientalis* has been observed in a 600m to 1930m altitudinal range [44]. It seems that its distribution is positively influenced by the presence of Acacia–Balanites vegetation and cracks in black cotton clay soil [23, 45], as observed in the Metema-Humera foci. In the Omo [14] and Awash [17] river valleys, and in the highlands of Belessa [30] and Libo Kemkem [46], black cotton soil was also claimed to form the habitat for *P. orientalis*.

Despite the ecological changes in the Metema and Humera foci, with Acacia–Balanites vegetation being substituted for crop agricultural schemes, *P. orientalis* is still the major vector of *L. donovani* [47]. Observation of male sandflies with unrotated genitalia suggests that deeply cracked black cotton soil is likely to be the most productive breeding habitat. Emergence from gaps in a stone wall in peri-domestic habitats [48] implicate that house wall material could serve the same function. This observation is further strengthened by the evidence from blood meal analysis: high numbers of blood fed, gravid and semi-gravid female *P. orientalis* were collected near villages despite the known high population density of sandflies in tickets of Acacia trees or farmland [49]. In the highlands of Belessa, no significant variation in populations size was noted between rainy and dry seasons [30], yet in the extra-domestic agricultural fields and thickets of A. seyal forests in Kafta-Humera, peak population was reached in March and April (dry season) [47].

While ecological differences have resulted in seasonality of the *P. orientalis* population size, geographical and ecological variation did not result in a difference in vector competence. Despite the distinct requirements for larval food and humidity during pupation, two colonies of *P. orientalis*; from a VL focus (1800–2000 m) and a non-endemic area (800 m), showed similar vector competence [50]. Furthermore, the colonies showed high similarity in morphology, transcriptomes, proteomes or enzymatic properties of the salivary components and mitochondrial genes. They interbred and the F1/F2 generations were shown to be even more fit in terms of reproductive capacity [50, 51].

Only few studies have described the natural feeding behaviour, peak of biting activity and blood meal preference of *P. orientalis* in Ethiopia. Ashford noted that except freshly blood-fed females, all stages of *P. orientalis* were observed to feed on plants with noxious sap. In terms of blood feeding, *P. orientalis* seem to be exophagic and catholic; in Metema-Humera up to 92 % (250/273) fed from cattle [49, 52], in Awash valley 54 % (51/94) fed from camels and 2 % (2/94) from squirrel and in Belessa 2 sandflies fed from porcupines [30]. According to the description of Ashford in his Belessa study, *P. orientalis* bit humans voraciously and was the most common species. Of 1018 dissected sandflies, 1006 were *P. orientalis* and most (900) were from human landing catch. Thus the lesser proportion of human blood meals in the other studies might indicate a zooprophylactic effect due to their sheer number. A study on peak human biting time of *P. orientalis* near Arbaya, Belessa district, documented that the peak was reached shortly after dark: between 28 °C and 19 °C and approximately 2:15 h after sunset [44]. Moreover, they were seen to come to bite man in large numbers in a gulley [30]. Also, it was indicated that mating occurs at dusk and wind considerably affects both the swarm size and mating behaviour of *P. orientalis*. The ubiquitousness of *P. orientalis*, its catholic feeding behaviour and phenotypic plasticity with no loss of vectorial competence may have resulted in its importance as a vector for *L. donovani* in Ethiopia.

### *Phlebotomus martini (Parrot) and Phlebotomus celiae* (*Minter)*

*Phlebotomus martini (P. martini)* was first described from Dire Dawa, Ethiopia; it has also been reported from Kenya, Somalia, Sudan, Uganda, and recently from Tanzania [53, 54]. It is incriminated as a vector of *L. donovani* in Sudan, Ethiopia, Kenya and Uganda [55]. The closely related *Phlebotomus celiae (P. celiae)* was described in ventilation shafts of termite hills of Kitui, Kenya, occurring in close association with *P. martini* [56]. Subsequently in 1984, *P. celiae* was reported in the Aba-Roba VL focus of Ethiopia, in a similar habitat [57]. *P. martini* has also been caught biting humans in the highlands of Belessa [30]. Additionally, *P. martini* has been caught in small numbers from the northern lowland foci [47] and the Rift valley [58]. It has so far been reported to exist at altitudes ranging from 637 to 1750 masl [36, 44, 47]. *P. martini* and *P. celiae* occur sympatrically in the south and southwest of Ethiopia. They are judged from abundance and infection rate data to be the primary and secondary agents of anthroponotic VL transmission respectively at an altitude of 1200 masl in this area [37]. From a study in Aba-Roba, it was documented that both species are nocturnal and exophagous. Their peak biting activity, as determined from human landing catches, was between 20:00 and 22:00 h both at termite hills and in dwelling compounds [36]. In terms of population dynamics in this focus, *P. martini* was abundant with no seasonal pattern while *P. celiae* populations reached their peak during the wet seasons (February to May and October to September), and were minimal during the dry season ( November to December and June to August) [36].

Though it is inferred that termite hills (*Macrotermes* termites) are the natural breeding and resting habitat for *P. martini* and *P. celiae*, no study has so far confirmed this in Ethiopia. Moreover, a survey in south-west Ethiopia, in the Omo and Akobo river basins and the drainage basin from Chow Bahar at an altitudinal range of 375 to 2000 masl (largely between 375 and 1000 masl), documented no association between leishmanin skin test positivity and the presence of both chimney and pipe organ type termite hills [14]. The study involved ecotypes occupied by different tribes: the Dassanetch; Nyangatom; Hamar; Suri; Bale; Anuak; Kerre; Shako; Ulam; Tishena, Bodi; Magugi; Bachada and Banna tribe, with little or no inter-tribal and/or external migration. Thus the existing data set underlines the possible existence of alternative resting and breeding sites that need to be explored. In addition, the blood meal source preference, vectorial competence and behaviour of both species in different ecological settings in Ethiopia (other than the Aba-Roba focus) is unknown.

### Aetiology of visceral leishmaniasis in Ethiopia

Deoxyribonucleic acid based typing of 63 new parasite isolates from VL cases from different parts of the country indisputably established that *L. donovani* is the causative agent of VL in Ethiopia [41, 59]. However, using 14 microsatellite markers, Gelanew et al. [41] differentiated between strains circulating in the north and south and subsequently, Zackay et al. [59] reached similar conclusions using HASPB (k26) PCR and sequencing. The aforementioned studies distinguished the circulating *L. donovani* in East Africa into two populations: the northern Ethiopia and the Sudan strain, and the southern Ethiopia and Kenya/Uganda strain. The difference between the two populations is congruent with a difference in sandfly vector (*P. orientalis vs P. martini/celiae*) [60]. The inheritance of genes responsible for virulence/drug-resistance depends on the extent of genetic recombination in the parasite. The intra-specific genetic exchange in natural Ethiopian *L. donovani* populations [61] and the co-existence of clonally and sexually reproducing strains in the southern foci [41] has recently been documented, indicating the existence of an enabling environment for genetic exchange. It was proposed that existing differences in clinical response to treatment may be due to parasite and/or host genetics. Yet, data from clinical trials in East Africa did not reflect a high genetic exchange potential and/or a difference in parasite genetics. A multicentre, open-label, randomized, controlled clinical trial compared three treatment regimens for VL with paromomycin (PM) monotherapy [62]. PM elicited the poorest treatment response at the Sudanese sites with final cure rates of 14.3 and 46.7 % at Um el Kher and Kassab respectively. The results in north Ethiopia (Gondar) were comparable to those in Kenya; the efficacy of PM was between 75 and 80 % respectively in both sites. In south Ethiopia (Arba Minch), PM showed a final cure rate of 96.6 %. However, in another trial comparing single dose AmBisome (liposomal amphotericin B) with multiple dose AmBisome (7 doses of 3 mg/kg) [63], the final cure rate in Sudan (Kassab) was 76 and 39 % for multiple and single (10 mg/kg) doses respectively, and similarly, in Ethiopia (Gondar) it was 71 % for multiple and 33 % for single (10mg/kg) doses, while in south Ethiopia (Arba Minch) the final cure rates were 100 % for multiple and single (10 mg/kg) doses. The presence of a large number of ethnic groups in south Ethiopia living in similar environmental and ecological conditions with no/little mixing and a more homogenous population in north Ethiopia implies that host and/or parasite genetics may not play an important role in the observed differences in treatment response. Intraregional differences in treatment response between East African sites can’t be fully explained by *L. donovani* variability. Thus, other than parasite and host genetics, socio-cultural conditions should be considered in planning VL control/elimination efforts with existing and future drugs in East Africa.

### Risk factors associated with VL disease or Leishmania infection in VL foci in Ethiopia

Many studies in Ethiopia, using either clinical VL or *Leishmania* infection as an end point, have implicated exogenic transmission as major source of infection both in the northern and southern foci. Early studies in the epidemiology of VL, using leishmanin skin test positivity as a read-out, documented a strong association of VL with age (increasing age), occupation (herding/night grading of cattle), and sex (more in male than females) [14, 23, 64, 65]. Entomological and epidemiological studies also support outdoor biting and sylvatic transmission as major source of infection [66]. Yet a recent study of clinical VL in children in north-west Ethiopia reported 23 % (28/122) of cases to be of the under five age group. Although it is impossible to rule out the possibility of outdoor biting, this might indicate the increasing risk of domestication of VL transmission in this focus [67]. In addition to the soil (black cotton soil) [14, 47], vegetation type (Acacia-Balanites vegetation) [39, 68], presence of termite hills [36, 37] and mass movement of people [22, 23, 32, 69], different behavioural, household and environmental factors have been implicated as risk factors for either asymptomatic *Leishmania* infection or clinical VL. Studies done in the Libo Kemkem and Fogera areas documented increasing age (per year), being male, sleeping outside at any time of the year, past history of VL in the family, living in a straw roofed house and whether the family owned sheep as risks for *Leishmania* infection [70]. The factors associated with clinical VL were dog ownership, sleeping under Acacia trees during the day, and habitually sleeping outside at night [39]. A similar study among residents of Kafta Humera incriminated family size (increased), living in a house with cracked walls, ownership of a goat and number of days spent in the farming fields as factors increasing the odds of having clinical VL [71]. In a similar area, another study comparing residents and migrant labourers concluded that sleeping under Acacia trees at night and lower educational status were associated with an increased risk of clinical VL in both populations. Living in a house with thatched walls and sleeping on the ground were associated with high risk of clinical VL for residents; among migrants those sleeping near dogs were most likely to have clinical VL [68]. A case–control study to understand the link between clinical VL and domestic animals screened dogs, cats, cattle, donkeys, sheep and goats. The outcome showed that dogs owned by people with a history of VL have a high risk of infection (OR:8.9, P:0.016) compared to cattle though the overall infection prevalence reported was a similar 42 % (18/43) and 40 % (36/90) in cattle and dogs respectively [40]. From studies so far, implicated domestic animals in VL transmission yet their role as risk and/or protective factors in specific eco-epidemiologic setting need to be ascertained.

Studies with respect to knowledge, attitude and practice and community acceptability of existing control measures are rare. A study in the rural communities of Libo Kemkem indicated that although the knowledge regarding signs, symptoms, and causes of VL was very low at the beginning of a longitudinal study, it improved substantially over a 3 year period, and the community had a positive attitude towards prevention [72]. A similar study in Addis Zemen town showed that in general good practice was low despite the good awareness and favourable attitude towards measures to prevent and control VL [73]. Overall, the studies implied a need to better understand the specific reasons for failure of effective community interventions and social mobilization.

Concomitant morbidities, especially HIV/VL co-infection have a strong impact on VL control. In 1995 only 7 HIV/VL co- infected cases were reported [74]. Subsequent hospital based studies in the northern foci, mainly serving migrant labourers, documented an alarming increase of HIV/VL co-infection: 29 % (167/375) [75], 41 % (87/212) [76], 38 % (92/241) [27] and 46.4 % (13/30) [77] in 2006, 2007 and 2010 respectively. More recently this trend is decreasing Mengesha et al. reported a lower prevalence of 10.4 % in 2014 [78]. Malnutrition is another important co-morbidity; a high prevalence of malnutrition (up to 95 %) was reported in VL patients [67, 78]. Intestinal parasitosis was also noted to be common in VL cases [67, 78]. Thus, in planning control efforts in the Ethiopian context, there is a need to consider HIV/VL co-infection, as well as parasitosis and/or malnutrition in VL patients as priorities.

### Risk mapping

**Fig. 3:**
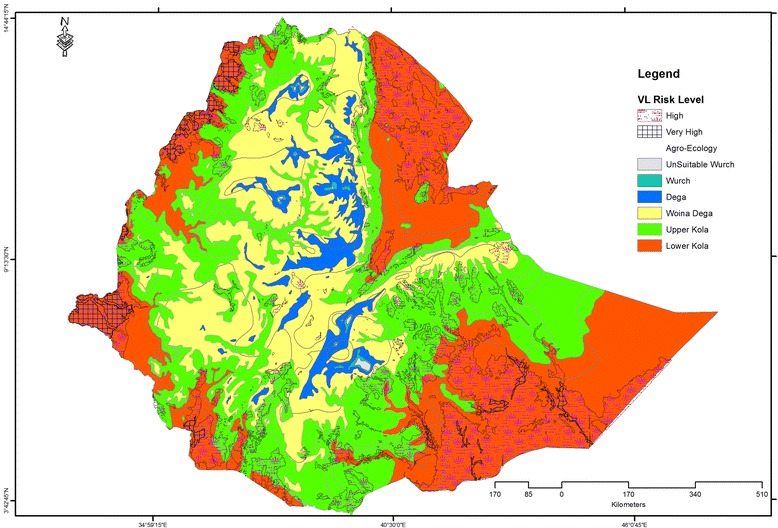
Visceral leishmaniasis risk areas in Ethiopia. The risk areas extend from the Kola to the Weina Dega agro-ecological zones; yet the major part of the high risk and very high risk areas are within the Kola (Lower and Upper) agro-ecological zone

Despite the long-standing presence and focalized distribution of VL in Ethiopia, little work has been done to determine its association with environmental factors. Understanding spatio-temporal diffusion patterns and the mapping of existing hotspots will have paramount importance for more focused interventions and control of the spread of VL. The use of techniques such as GIS and remote sensing (RS) will enable the analysis of complex information as well as geographically targeted control programmes. Good models, based on high quality predictor data of epidemiological and biological relevance, have reasonable accuracy in explaining the actual situation and are independently verifiable. A VL risk model for Ethiopia based on soil type, altitude, mean annual rainfall, surface temperature and slope vis-à-vis GPS data on clinical VL presence and absence predicted that 33 % of the total land mass is at high and very high risk of VL endemicity [8]. According to this model, over 3.2 million people live in areas at risk (Fig. 3), yet considering the large scale immigration of temporary labourers and settlers to these areas this might be an underestimation. Among the studied environmental factors, mean annual surface temperature (26–31 °C) and soil type (presence of vertisol) were the best predictors of VL transmission. The validation of the model through verification of the presence/absence of VL cases (random selection) showed an accuracy of 86 %. This modelling exercise provided important background information for a further study, which considered temporal information and additional environmental and climatic variables and focused on the Kafta Humera foci. Taking mean monthly rainfall and temperature, seasonal normalized difference vegetation index, soil type, altitude and slope, and comparing these to 15 years (1998 to 2012) of monthly VL data from the Kahsay Abera hospital, concluded that VL incidence and presence of transmission were directly related to temperature and presence of vertisol. The relation with altitude, rainfall, slope and the mean seasonal normalized difference vegetation index was inverse [79]. In terms of risk, the villages Bereket, Rawoyan, Baeker, Adebay, May Kadra and Humera town all were high risk areas for VL.

Regarding sandfly vectors, Gebre-Michael et al. [80] used environmental variables and historical sandfly data to make GIS based predictive maps of *P. orientalis* and *P. martini*. Their findings showed that while average altitude (12–1900 m), average annual mean temperature (15–30 °C), annual rainfall (274–1212 mm), average annual potential evapotranspiration (1264–1938 mm) and readily available soil moisture (62–113 mm) were found to be the best fitting ecological variables for *P. martini* presence; for *P. orientalis,* average altitude (200–2200 m), annual rainfall (180–1050 mm), annual mean temperature (16–36 °C) and readily available soil moisture (67–108 mm) were the major ecological determinants. On the other hand, mid-day land surface temperature (LST), dry season composite of the satellite data, average altitude, mean annual temperature and readily available soil moisture were the best ecological determinants for *P. martini. F*or *P. orientalis,* LST annual composite was the only important ecological determinant. According to this model the ecological extent of both vectors correspond with the climatic conditions currently prevailing and the lack of association with soil type might be due to ecological changes, for the sandfly data used were very old. Thus updating sandfly collection, soil, other environmental and remote sensing data to remodel the *P. martini* and *P. orientalis* distribution could help to better understand the (re)emergence of VL in Ethiopia.

## Conclusions

Visceral leishmaniasis, historically known to be a problem in the Kola area, has recently been spreading to the adjunct Woina Dega zones. The existing major agricultural schemes, ongoing and planned mega-projects such as sugar industries and sugar cane farming, dams for electricity/irrigation purposes and expansion of irrigation and/or rain fed agriculture both by small scale farmers and large scale investors all exist within the lower Kola to Weina Dega agro-ecological zones. Agriculture dominates Ethiopia’s economy; it contributes to 85 % of the employment, 45 % of the total GDP, 86 % of the total foreign earning and 70 % of the raw material requirement of local industries. Thus, the (re)emergence of VL in Ethiopia will not only have public health and social implications but also will have a direct impact on the country’s economy and further development. Yet, we conclude in our review that existing data seem incomplete to plan for a cost-effective and efficient VL control strategy. From the point of view of vector control, there is a need for updating the sandfly mapping and data on vector behaviour in specific ecosystems (resting, breading, biting and host preference). Transmission dynamics (domestic, peridomestic or sylvatic) and the role of domestic animals need to be ascertained. Additionally, the effectiveness and social acceptability of available vector control tools need be evaluated. Furthermore, looking into vector species implicated in the zoonosis of *L. donovani* in East Africa using appropriate trapping techniques might lead to the identification of a possible animal reservoir, or ascertain the absence of zoonosis in Ethiopia. In order to prevent the (re)emergence and spread of VL to areas adjacent to VL foci we suggest small scale spatio-temporal mapping. This enables the identification of hotspots and creates an understanding of their seasonality whereas the delineation of autochthonous transmission zones from the geographic extent of the sandfly habitat will enable mapping of areas at high risk of VL (re) introduction. Successful roll-out of prevention measures is only possible with reliable data on risk factors and the knowledge, aptitude and practice of the affected communities, though the few data available indicate that these are in favour of protective measures although the knowledge about VL is generally low. Thus, well designed studies identifying risk factors for exposure to infection and clinical VL, as well as a better understanding of knowledge, aptitude and practice are necessary. To reduce VL mortality, existing data underline the importance of the provision of supplementary nutrition, de-worming and appropriate care and treatment for HIV/VL.

## References

[1] Alvar J, Yactayo S, Bern C (2006). Leishmaniasis and poverty. Trends Parasitol.

[2] Alvar J, Velez ID, Bern C, Herrero M, Desjeux P, Cano J, Jannin J, den Boer M, Team WHOLC (2012). Leishmaniasis worldwide and global estimates of its incidence. PLoS One.

[3] Prajapati V, Mehrotra S. Advances in the Diagnosis of Visceral Leishmaniasis. J Mol Biomark Diagn. 2013;4:e118. doi:10.4172/2155-9929.1000e118.

[4] Hailu A, Gramiccia M, Kager PA (2009). Visceral leishmaniasis in Aba-Roba, south-western Ethiopia: prevalence and incidence of active and subclinical infections. Ann Trop Med Parasitol.

[5] Khalil EA, Zijlstra EE, Kager PA, El Hassan AM (2002). Epidemiology and clinical manifestations of Leishmania donovani infection in two villages in an endemic area in eastern Sudan. Trop Med Int Health.

[6] Bern C, Haque R, Chowdhury R, Ali M, Kurkjian KM, Vaz L, Amann J, Wahed MA, Wagatsuma Y, Breiman RF (2007). The epidemiology of visceral leishmaniasis and asymptomatic leishmanial infection in a highly endemic Bangladeshi village. Am J Trop Med Hyg.

[7] FMOH (2013). Guideline for Diagnosis, Treatment and Prevention of Leishmaniasis in Ethiopia. Edited by Unit NTD, Second edn.

[8] Tsegaw T, Gadisa E, Seid A, Abera A, Teshome A, Mulugeta A, Herrero M, Argaw D, Jorge A, Aseffa A (2013). Identification of environmental parameters and risk mapping of visceral leishmaniasis in Ethiopia by using geographical information systems and a statistical approach. Geospat Health.

[9] ANDERSON TF (1943). Kala-azar in the East African Forces. East Afr Med J.

[10] Coles A, Consgrove P, Robinson G. A preliminary report of an outbreak of Kala-Azar in a battalion of King’s African rifles. Transactions of the Royal Society of Tropical Medicine and Hygiene 1942, 36(1):25.

[11] Ayele T, Ali A (1984). The distribution of visceral leishmaniasis in Ethiopia. Am J Trop Med Hyg.

[12] Lindtjorn B (1984). Kala-azar in south-west Ethiopia: seasonal variation in disease occurrence. Trans R Soc Trop Med Hyg.

[13] Fuller GK, Lemma A, Gemetchu T (1974). Kula-azar in Southwest Ethiopia. Trans R Soc Trop Med Hyg.

[14] Fuller GK, Lemma A, Haile T, Gemeda N (1979). Kala-azar in Ethiopia: survey of south-west Ethiopia. The Leishmanin skin test and epidemiological studies. Ann Trop Med Parasitol.

[15] Fuller GK, DeSole G, Lemma A (1976). Kala-azar in Ethiopia: survey and leishmanin skin test results in the middle and lower Awash River Valley. Ethiop Med J.

[16] Ali A (1997). Leishmaniases survey in the Awash Valley: leishmanin skin test profile in the Upper Awash and surrounding areas. Ethiop Med J.

[17] Gemetchu T, Fuller GK (1976). Kala-azar in Ethiopia: the phlebotomid sandflies of the Awash River Valley. Ethiop Med J.

[18] Hailu A, Berhe N, Yeneneh H (1996). Visceral leishmaniasis in Gambela, western Ethiopia. Ethiop Med J.

[19] Hans H (1998). Agroecological Belts of Ethiopia Explanatory Notes on Three Maps at A Scale of 1:1,000,000.

[20] Ali A, Ashford RW (1994). Visceral leishmaniasis in Ethiopia. IV. Prevalence, incidence and relation of infection to disease in an endemic area. Ann Trop Med Parasitol.

[21] Lindtjorn B (1987). Visceral leishmaniasis in the Dawa valley, south Ethiopia. Ethiop Med J.

[22] Marlet MV, Sang DK, Ritmeijer K, Muga RO, Onsongo J, Davidson RN (2003). Emergence or re-emergence of visceral leishmaniasis in areas of Somalia, north-eastern Kenya, and south-eastern Ethiopia in 2000–01. Trans R Soc Trop Med Hyg.

[23] Fuller GK, Lemma A, Haile T, Atwood CL (1976). Kala-azar in Ethopia I: Leishmanin skin test in Setit Humera, a kala-azar endemic area in northwestern Ethopia. Ann Trop Med Parasitol.

[24] Mengesha B, Abuhoy M (1978). Kala-azar among labour migrants in Metema-Humera region of Ethiopia. Trop Geogr Med.

[25] McGregor A (1998). WHO warns of epidemic leishmania. Lancet.

[26] Lyons S, Veeken H, Long J (2003). Visceral leishmaniasis and HIV in Tigray, Ethiopia. Trop Med Int Health.

[27] Hurissa Z, Gebre-Silassie S, Hailu W, Tefera T, Lalloo DG, Cuevas LE, Hailu A (2010). Clinical characteristics and treatment outcome of patients with visceral leishmaniasis and HIV co-infection in northwest Ethiopia. Trop Med Int Health.

[28] ter Horst R, Collin SM, Ritmeijer K, Bogale A, Davidson RN (2008). Concordant HIV infection and visceral leishmaniasis in Ethiopia: the influence of antiretroviral treatment and other factors on outcome. Clin Infect Dis.

[29] Abbasi I, Aramin S, Hailu A, Shiferaw W, Kassahun A, Belay S, Jaffe C, Warburg A (2013). Evaluation of PCR procedures for detecting and quantifying Leishmania donovani DNA in large numbers of dried human blood samples from a visceral leishmaniasis focus in Northern Ethiopia. BMC Infect Dis.

[30] Ashford RW, Hutchinson MP, Bray RS (1973). Kala-azar in Ethiopia: Epidemiological studies in a highland valley. Ethiop Med J.

[31] Herrero M, Orfanos G, Argaw D, Mulugeta A, Aparicio P, Parreno F, Bernal O, Rubens D, Pedraza J, Lima MA (2009). Natural history of a visceral leishmaniasis outbreak in highland Ethiopia. Am J Trop Med Hyg.

[32] Alvar J, Bashaye S, Argaw D, Cruz I, Aparicio P, Kassa A, Orfanos G, Parreno F, Babaniyi O, Gudeta N (2007). Kala-azar outbreak in Libo Kemkem, Ethiopia: epidemiologic and parasitologic assessment. Am J Trop Med Hyg.

[33] Sordo L, Gadisa E, Custodio E, Cruz I, Simon F, Abraham Z, Moreno J, Aseffa A, Tsegaye H, Nieto J (2012). Low prevalence of Leishmania infection in post-epidemic areas of Libo Kemkem, Ethiopia. Am J Trop Med Hyg.

[34] Killick-Kendrick R (1999). The biology and control of phlebotomine sand flies. Clin Dermatol.

[35] Maroli M, Feliciangeli MD, Bichaud L, Charrel RN, Gradoni L (2013). Phlebotomine sandflies and the spreading of leishmaniases and other diseases of public health concern. Med Vet Entomol.

[36] Gebre-Michael T, Lane RP, Frame IA, Miles MA (1993). Leishmania donovani infections in phlebotomine sandflies from the kala-azar focus at Aba Roba in Ethiopia: DNA probe compared with conventional detection methods. Med Vet Entomol.

[37] Gebre-Michael T, Lane RP (1996). The roles of Phlebotomus martini and P.celiae (Diptera: Phlebotominae) as vectors of visceral leishmaniasis in the Aba Roba focus, southern Ethiopia. Med Vet Entomol.

[38] Kassahun A, Sadlova J, Dvorak V, Kostalova T, Rohousova I, Frynta D, Aghova T, Yasur-Landau D, Lemma W, Hailu A (2015). Detection of Leishmania donovani and L. tropica in Ethiopian eild rodents. Acta Trop.

[39] Bashaye S, Nombela N, Argaw D, Mulugeta A, Herrero M, Nieto J, Chicharro C, Canavate C, Aparicio P, Velez ID (2009). Risk factors for visceral leishmaniasis in a new epidemic site in Amhara Region, Ethiopia. Am J Trop Med Hyg.

[40] Kenubih A, Dagnachew S, Almaw G, Abebe T, Takele Y, Hailu A, et al. Preliminary survey of domestic animal visceral leishmaniasis and risk factors in north west Ethiopia. Tropical medicine & international health: TM & IH 2014. 10.1111/tmi.12418 25327874

[41] Gelanew T, Kuhls K, Hurissa Z, Weldegebreal T, Hailu W, Kassahun A, Abebe T, Hailu A, Schonian G (2010). Inference of population structure of Leishmania donovani strains isolated from different Ethiopian visceral leishmaniasis endemic areas. PLoS Negl Trop Dis.

[42] Elnaiem DE, Hassan HK, Osman OF, Maingon RD, Killick-Kendrick R, Ward RD (2011). A possible role for Phlebotomus (Anaphlebotomus) rodhaini (Parrot, 1930) in transmission of Leishmania donovani. Parasit Vectors.

[43] Alves AS, Mouta-Confort E, Figueiredo FB, Oliveira RV, Schubach AO, Madeira MF (2012). Evaluation of serological cross-reactivity between canine visceral leishmaniasis and natural infection by Trypanosoma caninum. Res Vet Sci.

[44] Ashford RW (1974). Sandflies (Diptra: Phlebotomidae) from Ethiopia: Taxonomic and Biological notes. J Med Entomol.

[45] Gematchu T, Zerihune A, Assefa G, Lemma A (1975). Observations on the sandfly (phlebotomidae) fauna of Setit Humera (Northwestern Ethiopia). Ethiop Med J.

[46] Gebre-Michael T, Balkew M, Alamirew T, Gudeta N, Reta M (2007). Preliminary entomological observations in a highland area of Amhara region, northern Ethiopia, with epidemic visceral leishmaniasis. Ann Trop Med Parasitol.

[47] Lemma W, Tekie H, Balkew M, Gebre-Michael T, Warburg A, Hailu A (2014). Population dynamics and habitat preferences of Phlebotomus orientalis in extra-domestic habitats of Kafta Humera lowlands--kala azar endemic areas in Northwest Ethiopia. Parasit Vectors.

[48] Moncaz A, Kirstein O, Gebresellassie A, Lemma W, Yared S, Gebre-Michael T, Hailu A, Shenker M, Warburg A (2014). Characterization of breeding sites of Phlebotomus orientalis - the vector of visceral leishmaniasis in northwestern Ethiopia. Acta Trop.

[49] Gebre-Michael T, Balkew M, Berhe N, Hailu A, Mekonnen Y (2010). Further studies on the phlebotomine sandflies of the kala-azar endemic lowlands of Humera-Metema (north-west Ethiopia) with observations on their natural blood meal sources. Parasit Vectors.

[50] Seblova V, Volfova V, Dvorak V, Pruzinova K, Votypka J, Kassahun A, Gebre-Michael T, Hailu A, Warburg A, Volf P (2013). Phlebotomus orientalis sand flies from two geographically distant Ethiopian localities: biology, genetic analyses and susceptibility to Leishmania donovani. PLoS Negl Trop Dis.

[51] Vlkova M, Sima M, Rohousova I, Kostalova T, Sumova P, Volfova V, Jaske EL, Barbian KD, Gebre-Michael T, Hailu A (2014). Comparative analysis of salivary gland transcriptomes of Phlebotomus orientalis sand flies from endemic and non-endemic foci of visceral leishmaniasis. PLoS Negl Trop Dis.

[52] Mamo H (1999). Production of Specific Antisera Against Selected Mammals and Identification of Blood Meals of Phlebotomine Sandflies Transmitting Visceral Leishmaniasis In Ethiopia.

[53] Lutomiah J, Omondi D, Masiga D, Mutai C, Mireji PO, Ongus J, Linthicum KJ, Sang R (2014). Blood meal analysis and virus detection in blood-fed mosquitoes collected during the 2006–2007 rift valley fever outbreak in Kenya. Vector Borne Zoonotic Dis.

[54] Clark JW, Kioko E, Odemba N, Ngere F, Kamanza J, Oyugi E, Kerich G, Kimbita E, Bast JD (2013). First report of the visceral leishmaniasis vector Phlebotomus martini (Diptera: Psychodidae) in Tanzania. J Med Entomol.

[55] Killick-Kendrick R (1990). Phlebotomine vectors of the leishmaniases: a review. Med Vet Entomol.

[56] Minter DM (1962). PHLEBOTOMUS (PHLEBOTOMUS) CELIAE SP. NOV. (DIPTERA, PSYCHODIDAE), A new Sandfly from Kenya. Ann Trop Med Parasitol.

[57] Ayele T, Mutinga MJ (1989). A new record of PHLEBOTOMUS CELIAE (DIPTERA, PHYCHODIDAE) In Ethiopia. Insect Sci Appllc.

[58] Balkew M, Gebre-Michael T, Berhe N, Ali A, Hailu A (2002). Leishmaniasis in the middle course of the Ethiopian Rift Valley: II. Entomological observations. Ethiop Med J.

[59] Zackay A, Nasereddin A, Takele Y, Tadesse D, Hailu W, Hurissa Z, Yifru S, Weldegebreal T, Diro E, Kassahun A (2013). Polymorphism in the HASPB repeat region of East African Leishmania donovani strains. PLoS Negl Trop Dis.

[60] Elnaiem DE (2011). Ecology and control of the sand fly vectors of Leishmania donovani in East Africa, with special emphasis on Phlebotomus orientalis. J Vector Ecol.

[61] Gelanew T, Hailu A, Schonian G, Lewis MD, Miles MA, Yeo M (2014). Multilocus sequence and microsatellite identification of intra-specific hybrids and ancestor-like donors among natural Ethiopian isolates of Leishmania donovani. Int J Parasitol.

[62] Hailu A, Musa A, Wasunna M, Balasegaram M, Yifru S, Mengistu G, Hurissa Z, Hailu W, Weldegebreal T, Tesfaye S (2010). Geographical variation in the response of visceral leishmaniasis to paromomycin in East Africa: a multicentre, open-label, randomized trial. PLoS Negl Trop Dis.

[63] Khalil EA, Weldegebreal T, Younis BM, Omollo R, Musa AM, Hailu W, Abuzaid AA, Dorlo TP, Hurissa Z, Yifru S (2014). Safety and efficacy of single dose versus multiple doses of Am Bisome for treatment of visceral leishmaniasis in eastern Africa: a randomised trial. PLoS Negl Trop Dis.

[64] Hailu A, Berhe N, Sisay Z, Abraham I, Medhin G (1996). Seroepidemiological and leishmanin skin test surveys of visceral leishmaniasis in south and southwest Ethiopia. Ethiop Med J.

[65] Ali A, Ashford RW (1994). Visceral leishmaniasis in Ethiopia. III. The magnitude and annual incidence of infection, as measured by serology in an endemic area. Ann Trop Med Parasitol.

[66] Ali A, Ashford RW (1993). Visceral leishmaniasis in Ethiopia. I. Cross-sectional leishmanin skin test in an endemic locality. Ann Trop Med Parasitol.

[67] Diro E, Lynen L, Gebregziabiher B, Assefa A, Lakew W, Belew Z, et al. Clinical aspects of paediatric visceral leishmaniasis in North-west Ethiopia. Tropical medicine & international health: TM & IH 2014. 10.1111/tmi.12407 25329449

[68] Argaw D, Mulugeta A, Herrero M, Nombela N, Teklu T, Tefera T, Belew Z, Alvar J, Bern C (2013). Risk factors for visceral Leishmaniasis among residents and migrants in Kafta-Humera, Ethiopia. PLoS Negl Trop Dis.

[69] Anema A, Ritmeijer K (2005). Treating HIV/AIDS and leishmaniasis coinfection in Ethiopia. CMAJ.

[70] Custodio E, Gadisa E, Sordo L, Cruz I, Moreno J, Nieto J, Chicharro C, Aseffa A, Abraham Z, Hailu T (2012). Factors associated with Leishmania asymptomatic infection: results from a cross-sectional survey in highland northern Ethiopia. PLoS Negl Trop Dis.

[71] Yared S, Deribe K, Gebreselassie A, Lemma W, Akililu E, Kirstein OD, Balkew M, Warburg A, Gebre-Michael T, Hailu A (2014). Risk factors of visceral leishmaniasis: a case control study in north-western Ethiopia. Parasit Vectors.

[72] Lopez-Perea N, Sordo L, Gadisa E, Cruz I, Hailu T, Moreno J, Aseffa A, Canavate C, Custodio E (2014). Knowledge, attitudes and practices related to visceral leishmaniasis in rural communities of Amhara State: a longitudinal study in northwest Ethiopia. PLoS Negl Trop Dis.

[73] Alemu A, Alemu A, Esmael N, Dessie Y, Hamdu K, Mathewos B, Birhan W (2013). Knowledge, attitude and practices related to visceral leishmaniasis among residents in Addis Zemen town, South Gondar, Northwest Ethiopia. BMC Public Health.

[74] Berhe N, Hailu A, Wolday D, Negesse Y, Cenini P, Frommel D (1995). Ethiopian visceral leishmaniasis patients co-infected with human immunodeficiency virus. Trans R Soc Trop Med Hyg.

[75] Ritmeijer K, Dejenie A, Assefa Y, Hundie TB, Mesure J, Boots G, den Boer M, Davidson RN (2006). A comparison of miltefosine and sodium stibogluconate for treatment of visceral leishmaniasis in an Ethiopian population with high prevalence of HIV infection. Clin Infect Dis.

[76] Mengistu G, Ayele B (2007). Visceral Leishmaniasis and HIV co-infection in patients admitted to Gondar university hospital, northwest Ethiopia. Ethiop J Health Dev.

[77] Hailu W, Weldegebreal T, Hurissa Z, Tafes H, Omollo R, Yifru S, Balasegaram M, Hailu A (2010). Safety and effectiveness of meglumine antimoniate in the treatment of Ethiopian visceral leishmaniasis patients with and without HIV co-infection. Trans R Soc Trop Med Hyg.

[78] Mengesha B, Endris M, Takele Y, Mekonnen K, Tadesse T, Feleke A, Diro E (2014). Prevalence of malnutrition and associated risk factors among adult visceral leishmaniasis patients in Northwest Ethiopia: a cross sectional study. BMC Res Notes.

[79] Solomon N (2014). Visceral Leishmaniasis (Kala-Azar) Risk Mapping using Geo-Spatial Tools: A Case Study in Kafta Humera District, North Western Ethiopia.

[80] Gebre-Michael T, Malone JB, Balkew M, Ali A, Berhe N, Hailu A, Herzi AA (2004). Mapping the potential distribution of Phlebotomus martini and P. orientalis (Diptera: Psychodidae), vectors of kala-azar in East Africa by use of geographic information systems. Acta Trop.

